# Characterization and Application of Guar Gum/Polyvinyl Alcohol-Based Food Packaging Films Containing Betacyanins from Pokeweed (*Phytolacca acinosa* Roxb.) Berries and Silver Nanoparticles

**DOI:** 10.3390/molecules28176243

**Published:** 2023-08-25

**Authors:** Xiaoqian Huang, Jiangfeng Song, Fengfeng Xu, Dawei Yun, Chenchen Li, Jun Liu

**Affiliations:** 1 College of Food Science and Engineering, Yangzhou University, Yangzhou 225127, China; mz120211966@stu.yzu.edu.cn (X.H.); mx120211237@stu.yzu.edu.cn (F.X.); dx120220228@stu.yzu.edu.cn (D.Y.); mz120201800@stu.yzu.edu.cn (C.L.); 2 Institute of Farm Product Processing, Jiangsu Academy of Agricultural Sciences, Nanjing 210014, China; songjiangfeng102@163.com

**Keywords:** pokeweed betacyanins, Ag nanoparticles, color-changing films, active packaging, smart packaging

## Abstract

Food packaging films were prepared by using guar gum/polyvinyl alcohol (GP) as the film matrix, 2% Ag nanoparticles (AgNPs) as reinforcing filler and antimicrobial agent, and 1%, 2% and 3% pokeweed betacyanins (PB) as the colorant and antioxidant agent. The structures and color-changing, barrier, mechanical, thermal and antioxidant/antibacterial properties of different films were measured. The results show that the PB were pH-sensitive pigments with pink, purple and yellow colors at pH 3–8, pH 9–11 and pH 12, respectively. PB improved the compatibility of guar gum and polyvinyl alcohol through hydrogen bonds. The films with PB showed a color-changing capacity under ammonia vapor and good color stability in chilled storage. AgNPs and PB elevated the barrier capacity of GP film to light, water vapor and oxygen gas. Meanwhile, AgNPs and PB improved the stiffness, thermal stability and antioxidant/antibacterial activity of GP film. The film with AgNPs and 3% PB showed the highest barrier capacity, stiffness, thermal stability and antioxidant/antimicrobial activity. In shrimp spoilage test, the films with AgNPs and 2% and 3% PB indicated shrimp freshness through film color changes. The results reveal the potential use of the prepared films in active and smart packaging.

## 1. Introduction

Petroleum-based packaging materials are widely used in the food industry due to their excellent mechanical property, gas/water resistance and low cost. However, petroleum-based packaging materials are non-renewable resources and non-biodegradable environmental pollutants [1]. Nowadays, the invention of biodegradable polymer-based packaging films is considered as a promising path to solve severe energy and environmental crises caused by petroleum-based packaging materials [2]. Natural polysaccharides and proteins isolated from animals, plants and microorganisms are good sources of biopolymers when preparing renewable and biodegradable food packaging materials [3].

Guar gum is one kind of non-ionic water-soluble polysaccharide isolated from the seed of *Cyamopsis tetragonolobus*, a drought-tolerant plant belonging to the Leguminosae family [4]. Structurally speaking, guar gum is a member of the galactomannans, with (1 → 4)-β-d-mannopyranosyl backbones and (1 → 6)-α-d-galactopyranosyl branch chains. The molecular ratio of galactose to mannose is about 1:2 in guar gum [5]. Considering guar gum is abundant in nature and has low cost, it shows application potentials in the cosmetic, food, pharmaceutical and environmental industries [6]. In the food industry, guar gum has been used as a food additive to improve the texture and taste of products [5]. Meanwhile, guar gum with a superior gelling and film-forming capacity is suitable for food packaging film inventions [7]. However, the neat guar gum films are too brittle and hydrophilic. In this respect, many strategies such as blending with other polymers and incorporating functional ingredients (e.g., polyvinyl alcohol, metal nanoparticles and plant extracts) have been designed to elevate the performance of guar gum films [7].

Polyvinyl alcohol is a non-toxic and water-soluble polymer with good mechanical properties, biodegradability and biocompatibility, and thus is widely used in food packaging films [8,9,10]. The hydroxyl groups present in the main backbone of polyvinyl alcohol are responsible for the strong intra- and inter-molecular hydrogen bonds with guar gum, endowing guar gum/polyvinyl alcohol film with increased mechanical properties but reduced hydrophilicity [11,12]. Metal nanoparticles, one type of nano-sized materials, have been widely used to elevate the performance of guar gum films [7]. Before now, different types of metal nanoparticles including Ag, ZnO and TiO_2_ nanoparticles have been added into guar gum-based films [12,13,14,15]. In active packaging field, guar gum-based films containing metal nanoparticles show good potentials in extending the shelf life of cheese [14] and strawberries [15]. In recent years, Ag nanoparticles (AgNPs) have been frequently used as reinforcing filler and antibacterial agents in guar gum-based films [12,15]. This is because AgNPs can interact with the guar gum matrix and show broad-spectrum antimicrobial properties. Despite this, the cytotoxicity of AgNPs cannot be ignored, where overdosing AgNPs might cause damage and toxicity by interacting with cell membranes [16]. Therefore, the contents of AgNPs in the packaging films must be closely controlled.

Recently, plant pigments, especially anthocyanins from red cabbage [17], red morningglory [11] and sumac [18], have been incorporated into guar gum-based films to endow the films with a unique pH-sensitive color-changing capacity. Meanwhile, anthocyanins can improve the barrier capacity and antioxidant activity of guar gum-based films [11,17,18]. Notably, the unique pH-sensitive color-changing capacity of guar gum-based films containing anthocyanins makes it possible for the films to indicate the freshness of chicken [11], pork and soybean milk [17] in smart packaging. However, betacyanins, another category of plant pigment, have not been used in inventing guar gum-based smart packaging films. Moreover, the packaging films developed by the combined use of guar gum, metal nanoparticles and betacyanins have not been reported yet.

Pokeweed (*Phytolacca acinosa* Roxb.), also called Shanglu in China, is a traditional herb that has been cultivated in China for thousands of years [19]. Pokeweed can produce red-violet berries, which are rich in betacyanins [20]. Nonetheless, betacyanins from pokeweed (*P. acinosa* Roxb.) berries have not been used in inventing smart packaging films up to now. Here, we developed food packaging films by using guar gum/polyvinyl alcohol as the film matrix, AgNPs as the reinforcing filler and antibacterial agent, and pokeweed betacyanins (PB) as the colorant and antioxidant agent. This study aimed to investigate the combined effects of AgNPs and PB on the performance of guar gum-based films.

## 2. Results and Discussions

### 2.1. pH-Sensitive Color-Changing Capacity of PB

**Figure 1:**
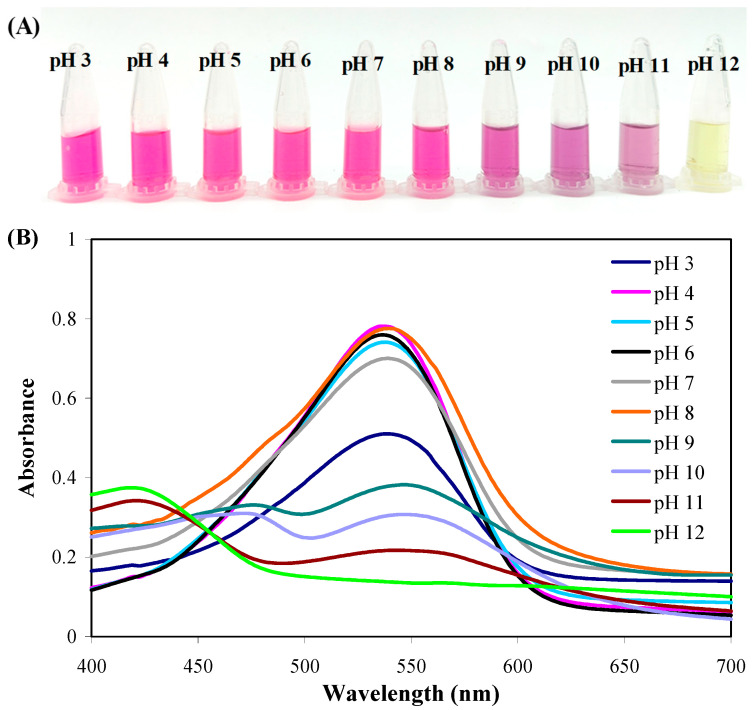
pH-sensitive color-changing capacity of PB powders. (**A**) Colors of PB solution with pH 3–12. (**B**) Visible spectra of PB solution with pH 3–12.

Previous studies have demonstrated that betacyanins from beet, pitaya, cactus, cockscomb and amaranth have pH-sensitive color-changing capacities [21,22]. In this study, betacyanins isolated from pokeweed berries were checked for their pH-sensitive color-changing capacity for the first time. As shown in Figure 1A, PB solutions presented pink, purple and yellow colors at pH 3–8, pH 9–11 and pH 12, respectively. The color-changing profiles of PB were different from those of betacyanins from beet [22], pitaya [23], cactus [24], cockscomb [21] and amaranth [25]. This was because betacyanins from different plants had different chemical compositions [22]. PB consisted of betanin 6′-*O*-sulfate and betanin, which were different from the chemical compositions of betacyanins from other plants [26]. From the visible spectra of PB solutions (Figure 1B), it could be seen that the maximum absorption peak of the solutions moved from 535 nm (pH 3–8) to 550 nm (pH 9–11) and even disappeared (pH 12). Similar changes were documented in the visible spectra of cactus betacyanins [24] and cockscomb betacyanins [21]. The color-changing capacity of PB was attributed to the degradation of betacyanins under alkaline conditions [27].

### 2.2. Structural Characterization of Films

**Figure 2:**
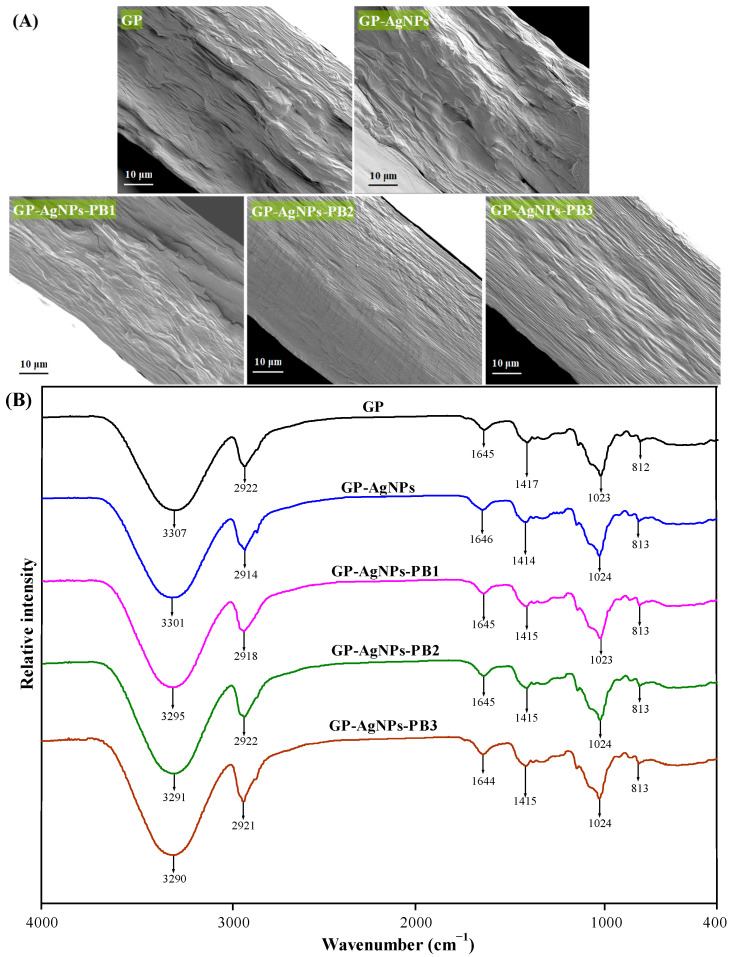
Structural characterization of films. (**A**) Cross-sectional morphology of GP, GP-AgNPs, GP-AgNPs-PB1, GP-AgNPs-PB2 and GP-AgNPs-PB3 films as observed by SEM. (**B**) Infrared spectra of GP, GP-AgNPs, GP-AgNPs-PB1, GP-AgNPs-PB2 and GP-AgNPs-PB3 films.

The inner microstructures of GP films with and without AgNPs and PB were checked via their cross-sections using SEM (Figure 2A). The GP film showed a heterogeneous and phase-separated morphology, indicating guar gum and polyvinyl alcohol had a low compatibility. The phase-separation in GP film was because guar gum had a higher viscosity and density than polyvinyl alcohol [28]. The GP-AgNPs film and GP-AgNPs-PB1 film presented similar cross-sections to the GP film, suggesting AgNPs and 1% PB did not significantly change the cross-sectional morphology. However, the GP-AgNPs-PB2 film and GP-AgNPs-PB2 film showed good homogeneity without obvious phase-separation. It was interfered that suitable amounts of PB (2% and 3%) formed stable interactions with guar gum/polyvinyl alcohol and improved their compatibility. Other studies also showed that pitaya betacyanins [28] and cockscomb betacyanins [21] elevated the homogeneity of phase-separated locust bean gum/polyvinyl alcohol films.

Intermolecular interactions among film components were checked by infrared spectra (Figure 2B). The infrared spectrum of the GP film was consistent with that in the literature [11,12,29]. The primary infrared bands of GP film were located at 3307, 2922, 1645, 1417 and 1023 cm^−1^, attributed to the O–H stretching of hydroxyl groups/the presence of moisture in the film, the C–H stretching of methylene groups, the O–H bending of associated water, the C–H bending of methylene groups, and the C–O stretching of sugar residues, respectively. GP-AgNPs film showed a similar infrared spectrum to the GP film, indicating weak coordination between AgNPs and GP film. A similar result was observed by Gasti et al. [12] in GP films containing in-situ-generated AgNPs. Compared with the GP-AgNPs film, GP-AgNPs-PB films showed a stronger O–H stretching band. The reason was that PB contained plenty of amino and hydroxyl groups that formed strong hydrogen bonds with GP film [21]. Also, the O–H stretching intensity of GP-AgNPs-PB films increased with an increasing amount of PB. The above results indicate that the PB formed stronger interactions with the GP film in comparison with AgNPs. The formation of PB–GP intermolecular hydrogen bonds promoted the fusion of guar gum and polyvinyl alcohol, which was consistent with the inner microstructure of GP-AgNPs-PB films (Figure 2A).

### 2.3. Color-Related Properties of Films

**Figure 3:**
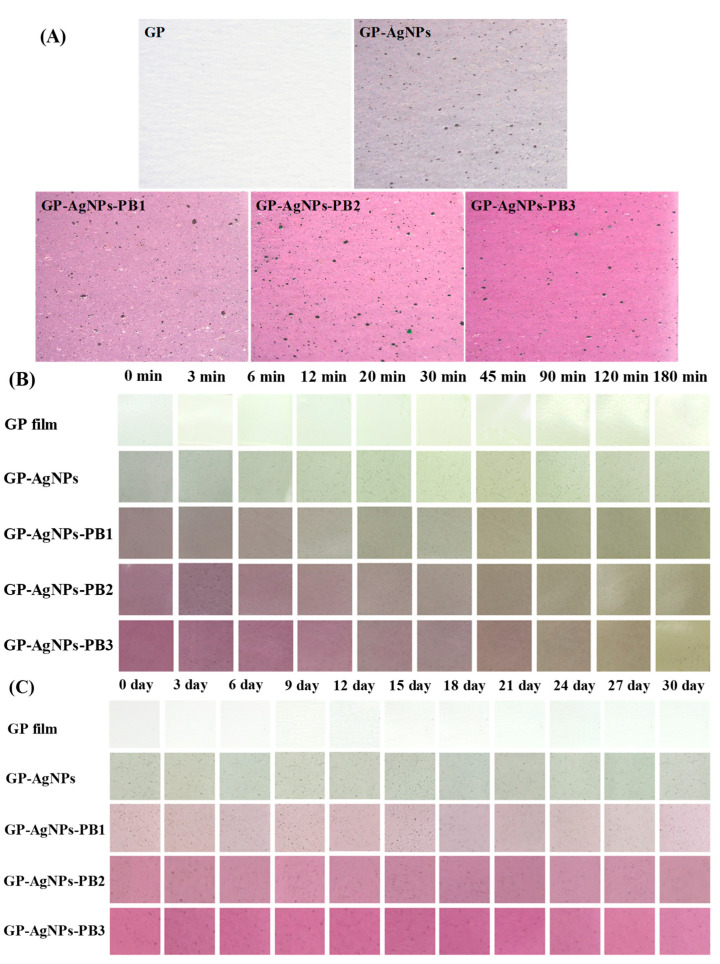
(**A**) Initial colors of GP, GP-AgNPs, GP-AgNPs-PB1, GP-AgNPs-PB2 and GP-AgNPs-PB3 films. (**B**) Color-changing capacity in ammonia vapor and (**C**) color stability of GP, GP-AgNPs, GP-AgNPs-PB1, GP-AgNPs-PB2 and GP-AgNPs-PB3 films during chilled storage.

The surface color of the films with and without AgNPs and PB was observed by video microscope. Figure 3A shows that the AgNPs made the colorless GP film gray, while PB gave the GP film a red-violet color. It was also noted that AgNPs were randomly distributed in the GP-AgNPs and GP-AgNPs-PB films. The color value measurement results in Table 1 further demonstrate the color changes of the GP film after adding AgNPs and PB. The brightness (L*) value of GP film was decreased by AgNPs and PB, while the redness (a*) value of GP film was increased by PB. Meanwhile, the a* value and Δ*E* value (total color difference) of GP-AgNPs-PB films gradually increased with an increasing PB amount, which agrees with the step-deepened red-violet color of GP-AgNPs-PB films (Figure 3A). A red-violet color was also noted in the films containing beet betacyanins [30] and pitaya betacyanins [31].

The color-changing capacity of the films was checked under ammonia vapor, which simulated volatile nitrogen released by rotten meat. As shown in Figure 3B, the GP film and GP-AgNPs film did not show color changes in ammonia vapor because these two films lacked color-changeable ingredients. However, GP-AgNPs-PB films gradually faded in ammonia vapor, due to betacyanins’ degradation under alkaline conditions [21]. Notably, GP-AgNPs-PB films with higher PB amounts showed lower fading rates. Previous studies also reported the films containing other betacyanins were much more sensitive to ammonia vapor [24,31]. The unique ammonia-sensitive property of GP-AgNPs-PB films was much more useful in smart packaging, especially for monitoring the volatile nitrogen released by rotten meat.

The capacity of the films to maintain their color stability during storage is very important for smart packaging. Figure 3C shows that all the films did not remarkably change their colors over 30 days of chilled storage. In this respect, all the films did not show eye-distinguishable color change [32]. Thus, it could be concluded that GP-AgNPs-PB films effectively maintained their colors during chilled storage and were suitable to be used in practical smart packaging. Recently, Yao et al. [28] also found that polyvinyl alcohol/locust bean gum films containing pitaya betacyanins effectively maintained their colors under chilled storage, which was because a low temperature is beneficial to maintaining the stability of betacyanins.

### 2.4. Barrier Properties of Films

**Figure 4:**
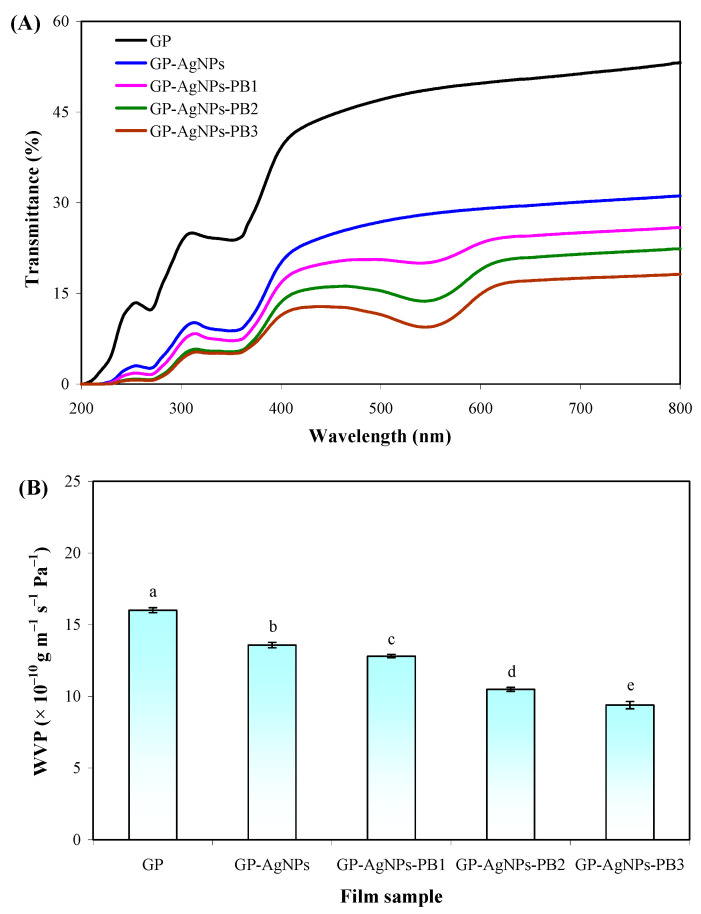

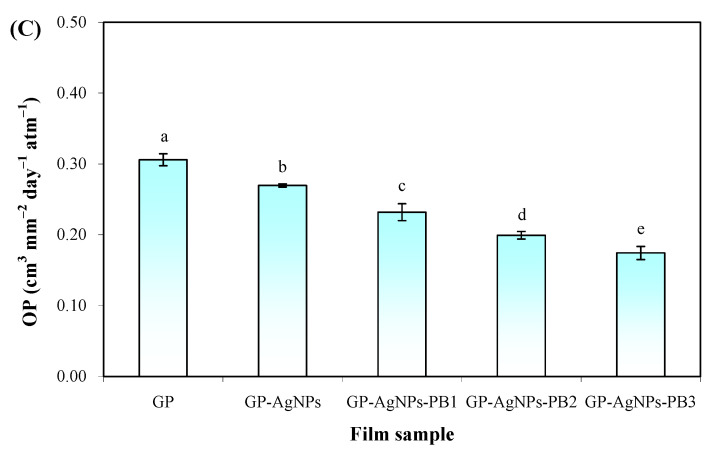
The barrier capacities of GP, GP-AgNPs, GP-AgNPs-PB1, GP-AgNPs-PB2 and GP-AgNPs-PB3 films. (**A**) UV-vis light barrier capacity. (**B**) The water vapor permeability. (**C**) The oxygen permeability. Values are given as mean ± SD (*n* = 6). Different lower case letters in the same figure indicate significant difference (*p* < 0.05).

The transmittance of the films with and without AgNPs and PB was tested in the UV-vis range (Figure 4A). the GP film showed a certain barrier capacity against UV-vis light, which was because the inner heterogeneous state of the GP film affected light refraction [28]. AgNPs significantly enhanced the light barrier capacity of GP film, associated with the light-scattering ability of AgNPs [7]. Gasti et al. [12] also found an enhanced light barrier capacity in GP film containing in-situ-generated AgNPs. On the basis of AgNPs, PB further elevated the light barrier capacity of the GP film. Different from AgNPs, PB exhibited a light barrier capacity through the chromophore in betacyanins. As reported, the double bonds (C=N, C=O and C=C) of betacyanins have a good light absorption ability [21]. It was noted that GP-AgNPs-PB films underwent transmittance reduction at 535 nm, which agrees with the absorption peak of PB in a neutral solution (Figure 1B). Among the films, the GP-AgNPs-PB3 film with the highest PB amount showed the lowest transmittance.

The WVP values of the films with and without AgNPs and PB were measured to evaluate their water vapor barrier capacity. Figure 4B shows that all the films had certain water vapor barrier capacities, which was because all the films had intact inner structures without any cracking (Figure 2A). Due to the hydrophilic nature of guar gum and polyvinyl alcohol, the GP film presented the highest WVP. Meanwhile, many water vapor passages existed in the phase-separated GP film matrix, making water vapor molecules more easily permeable within the GP film [29]. GP-AgNPs film showed a 15.24% decrease in WVP, compared to GP film. This was because AgNPs had a hydrophobic nature and possessed a low affinity for water vapor [7]. Gasti et al. [12] also reported that the in-situ-generated AgNPs increased the water vapor barrier capacity of GP film. The WVP of the GP-AgNPs film was further decreased by PB. Meanwhile, GP-AgNPs-PB films showed a significant WVP-decreasing trend with increasing PB amounts. It was inferred that PB formed strong hydrogen bonds with guar gum and polyvinyl alcohol, which restricted the interactions of the film matrix and water vapor [23]. As compared to the GP film, the GP-AgNPs-PB2 film and GP-AgNPs-PB3 film showed 34.48% and 41.29% WVP reductions, respectively. This was consistent with the homogeneous and compact structures of GP-AgNPs-PB2 and GP-AgNPs-PB3 films (Figure 2A). Similarly, Akhila et al. [11] found *Ipomoea coccinea* anthocyanins dose-dependently decreased the WVP of GP film. 

The OP values of the films with and without AgNPs and PB are shown in Figure 4C, revealing their oxygen barrier capacity. The OP of the films showed the same trend as their WVP (Figure 4B). The OP of the GP film was reduced by AgNPs, which was because the impermeable AgNPs acted as fillers and decreased the available oxygen transport volume in the film matrix [33]. The GP-AgNPs-PB films showed lower OP values than the GP-AgNPs film, indicating that PB further decreased the OP of the films. Notably, the OP values of GP-AgNPs-PB films were negatively correlated with PB amount. Ge and Popham [34] suggested the OP of packaging films containing betacyanins was affected by the films’ inner structures, where the films with more compact structures showed a lower OP. In this respect, the lower OP of the GP-AgNPs-PB2 and GP-AgNPs-PB3 films was associated with their more compact structures (Figure 2A).

### 2.5. Mechanical Properties of Films

**Figure 5:**
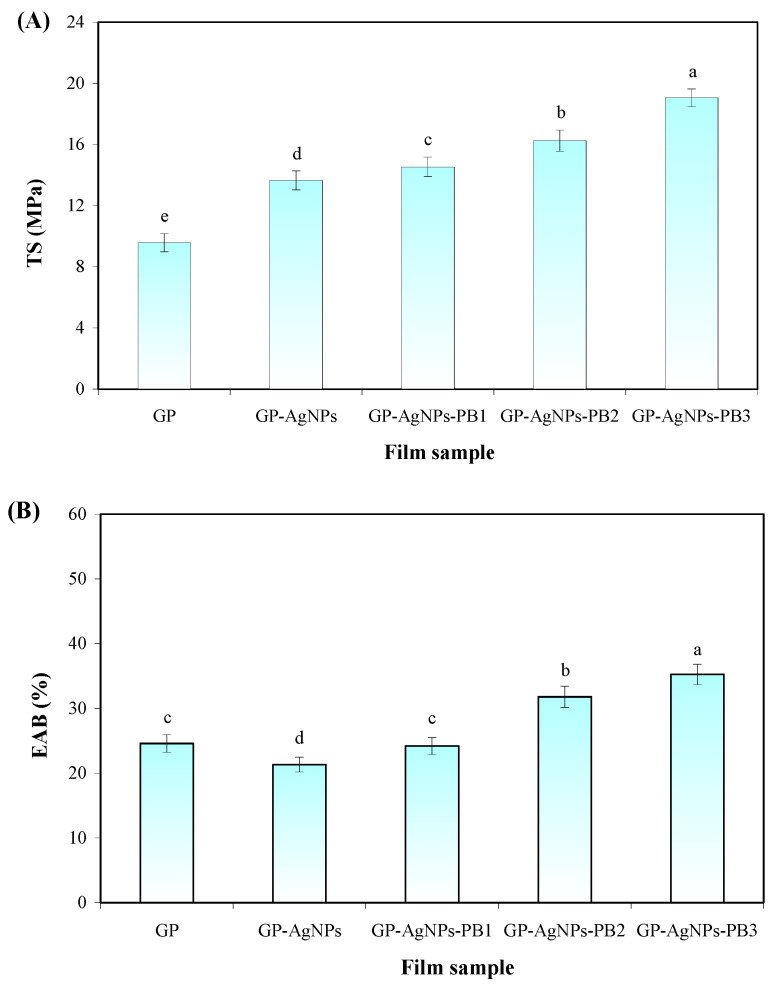
Mechanical properties of films. (**A**) TS of GP, GP-AgNPs, GP-AgNPs-PB1, GP-AgNPs-PB2 and GP-AgNPs-PB3 films. (**B**) EAB of GP, GP-AgNPs, GP-AgNPs-PB1, GP-AgNPs-PB2 and GP-AgNPs-PB3 films. Values are given as mean ± SD (*n* = 6). Different lower case letters in the same figure indicate significant difference (*p* < 0.05).

The mechanical properties of films with and without AgNPs and PB were reflected by their TS and EAB (Figure 5). The lowest TS and EAB values were noted in the GP film, suggesting the film had the worst stiffness and flexibility. The weak mechanical properties of the GP film are due to the incompatibility of guar gum and polyvinyl alcohol that restricted polymeric chain interaction and cohesion (Figure 2A). AgNPs increased the TS but decreased the EAB of the GP film. This was because AgNPs acted as reinforcing fillers and produced a stiffer film. At the same time, AgNPs reduced the association of polymeric chains and produced a less flexible film [33]. Similar trends were found in guar gum-based films with different metal nanoparticles [15,33]. GP-AgNPs-PB films had higher TS and EAB values than the GP-AgNPs film, indicating the stiffness and flexibility of the film were elevated by PB. This was because PB had strong hydrogen bonds with the GP matrix and greatly improved the compatibility of guar gum and polyvinyl alcohol (Figure 2A). As a result, the polymeric chain interaction and cohesion were greatly enhanced by PB. Other researchers also demonstrated that plant betacyanins could increase the TS and EAB of polymer-based films [21,23]. Interestingly, the TS and EAB of GP-AgNPs-PB films increased with an increasing PB amount, which is consistent with the formation of homogeneous and compact structures in GP-AgNPs-PB2 and GP-AgNPs-PB3 films (Figure 2A).

### 2.6. Thermal Property of Films

**Figure 6:**
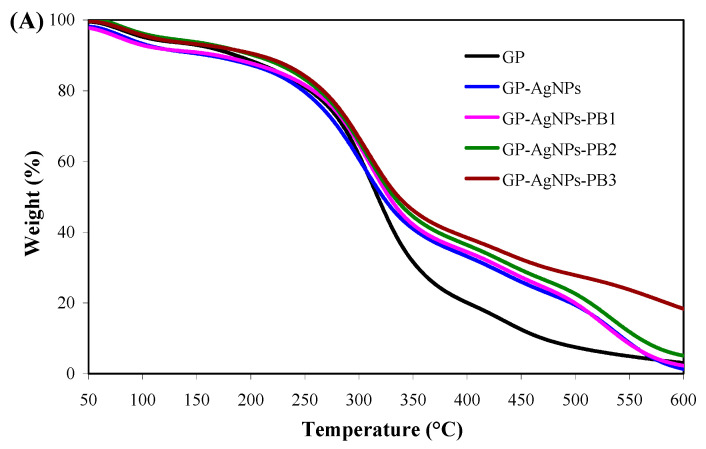

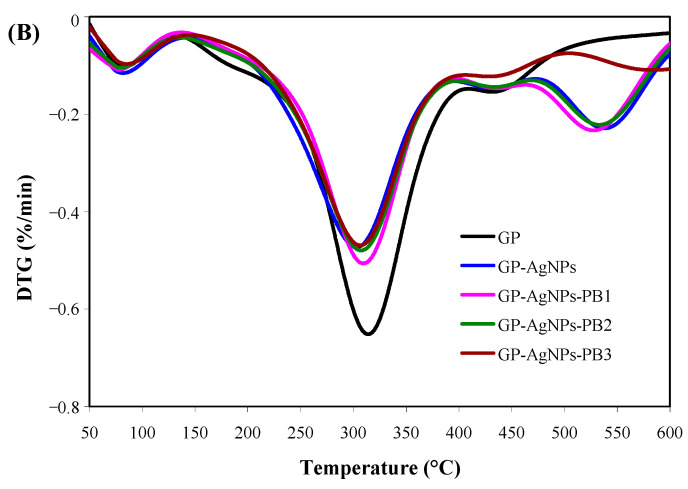
Thermal properties of films. (**A**) TGA curves of GP, GP-AgNPs, GP-AgNPs-PB1, GP-AgNPs-PB2 and GP-AgNPs-PB3 films. (**B**) DTG curves of GP, GP-AgNPs, GP-AgNPs-PB1, GP-AgNPs-PB2 and GP-AgNPs-PB3 films.

The thermal property of the films with and without AgNPs and PB was determined by thermogravimetric analysis (TGA). The thermal degradation of the films could be divided into three mass loss processes according to TGA and the derivative thermogravimetric (DTG) curves (Figure 6). The first mass loss was caused by moisture loss at 50–145 °C [11]. The second mass loss was due to the decomposition of small molecules (e.g., glycerol and PB) and the breakdown of polymeric chains at 145–410 °C [30]. The last mass loss was caused by the oxidative decomposition of the remaining carbon after 410 °C [12]. The main thermal degradation of the films was noted at the second mass loss process, with the fastest degradation occurring at 313–321 °C (Figure 6B). Overall, the GP film degraded faster than the GP-AgNPs and the GP-AgNPs-PB films, suggesting AgNPs and PB both elevated thermal stability. Gasti et al. [12] also found thermal stability was increased by the in-situ-generated AgNPs in the GP film, which was implied by the reduced free volume of film matrix. Notably, the GP-AgNPs-PB films showed higher thermal stability than the GP-AgNPs film. The thermal stability of GP-AgNPs-PB films increased with increasing PB amount. The increased thermal stability in GP-AgNPs-PB films resulted from the improved compatibility of guar gum and polyvinyl alcohol (Figure 2A) and the hydrogen bonds between the PB and film matrix (Figue 2B). The elevated thermal stability in GP-AgNPs-PB films effectively supported the strong barrier properties (Figure 4) and mechanical properties (Figure 5) of these films. In previous studies, an improved thermal stability was also noted in the films containing beet betacyanins [30] and pitaya betacyanins [31].

### 2.7. Antioxidant Activity of Films

**Figure 7:**
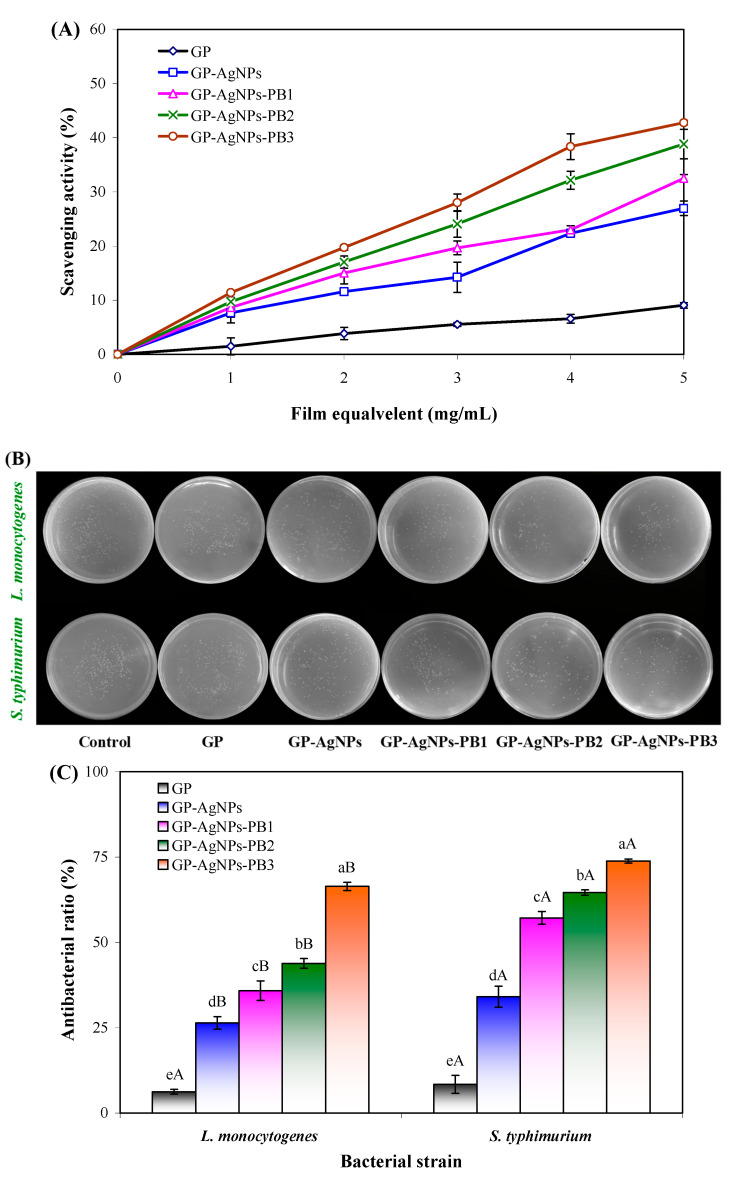
(**A**) DPPH radical-scavenging activity of GP, GP-AgNPs, GP-AgNPs-PB1, GP-AgNPs-PB2 and GP-AgNPs-PB3 films. (**B**) Antimicrobial performance of GP, GP-AgNPs, GP-AgNPs-PB1, GP-AgNPs-PB2 and GP-AgNPs-PB3 films. (**C**) Antibacterial activity of GP, GP-AgNPs, GP-AgNPs-PB1, GP-AgNPs-PB2 and GP-AgNPs-PB3 films. Values are given as mean ± SD (*n* = 3). Different upper case letters indicate a statistically significant difference in antibacterial activity (*p* < 0.05) within the same film against different bacterial strains. Different lower case letters indicate statistically significant differences in antibacterial activity (*p* < 0.05) among different films against the same bacterial strain.

The films with and without AgNPs and PB were judged by their DPPH-scavenging effect (Figure 7A), where the GP film had the worst DPPH-scavenging effect. This agreed with the report of Akhila et al. [11], who also found that the GP film had a negligible DPPH-scavenging effect. The GP-AgNPs film showed a 197.68% scavenging effect improvement at 5 mg/mL, compared to GP film. The reason was that AgNPs could capture free radicals by accepting electrons [35]. GP-AgNPs-PB films had 258.43−372.30% scavenging effect improvements at 5 mg/mL, compared to the GP film. At the same time, the scavenging effects of GP-AgNPs-PB films were positively correlated with the PB amount. These results reveal that the antioxidant activity was further increased by PB, which was because betacynians had phenolic hydroxyl groups and were able to donate hydrogen to DPPH radicals [21]. Yao et al. [22] also demonstrated betacynians from different plants elevated the DPPH-scavenging effect of starch-based films. The GP-AgNPs and GP-AgNPs-PB films with antioxidant activity were beneficial to preventing food oxidation in practical applications.

### 2.8. Antimicrobial Activity of Films

*L. monocytogenes* and *S. typhimurium* are common foodborne bacteria; *S. typhimurium* is Gram-negative and *L. monocytogenes* is Gram-positive. Hence, these two bacteria were chosen for the antimicrobial assay in this study. The antibacterial activity of the films with and without AgNPs and PB was tested by the colony counting method. Figure 7B,C shows that the GP film did not have an obvious antimicrobial activity, which agrees with the finding of Gasti et al. [12]. AgNPs significantly elevated the antimicrobial ratio of the GP film against *L. monocytogenes* and *S. typhimurium* to 26.39% and 34.07%, respectively. The antibacterial activity of AgNPs was attributed to their ultra-fine size and large surface area, through which AgNPs could easily penetrate the cell membrane and damage intracellular bio-molecules [36]. The antibacterial activity of GP-AgNPs film was further elevated by PB. Meanwhile, the antibacterial ratio of GP-AgNPs-PB films increased with an increasing PB amount. The maximum antibacterial ratio was noted in the GP-AgNPs-PB3 film, reaching 66.41% and 73.81% for *L. monocytogenes* and *S. typhimurium*, respectively. Several previous studies also reported the antibacterial activities of the films were increased by betacyanins [24,25,37]. Despite this, the detailed antibacterial mechanisms of betacyanins are still unclear. It was interfered the antibacterial activity of betacyanins might be associated with the increased cellular membrane permeability [37]. Interestingly, GP-AgNPs and GP-AgNPs-PB films showed stronger antibacterial activity against *S. typhimurium*. This was because Gram-negative *S. typhimurium* had a thinner cell wall than Gram-positive *L. monocytogenes* [25]. The above findings suggest GP-AgNPs-PB films could be used in active packaging to prevent microbial contamination.

### 2.9. Application of Films in Indicating the Quality of Shrimp

Fresh shrimp, due to endogenous protein decomposition and microbial action, is highly perishable and has a short shelf life. Therefore, it is important for customers and sellers to know the real-time quality of shrimp. In recent years, smart packaging films with betacyanins have been demonstrated as one type of convenient and non-destructive tool to indicate the real-time quality of shrimp [27]. In this study, the capacity of the films to indicate the quality of shrimp was determined. Table 2 shows that only GP-AgNPs-PB2 and GP-AgNPs-PB3 films underwent obvious color changes during shrimp storage. However, the GP-AgNPs-PB1 film did not undergo obvious color changes because of the low PB amount. The color changes of GP-AgNPs-PB2 and GP-AgNPs-PB3 films were caused by the volatile ammonia compounds released by shrimp [21]. To know the real quality of shrimp, shrimp TVB-N level (a reliable index for evaluating aquatic product quality) was detected. Table 2 shows that the TVB-N was greater than the fresh limit (20 mg/100 g) at 32 h, reaching 23.22 mg/100 g. The real-time color of GP-AgNPs-PB2 and GP-AgNPs-PB3 films at 32 h was dark red, which differed from the colors of these two films at 0–24 h. Therefore, GP-AgNPs-PB2 and GP-AgNPs-PB3 films were considered as promising intelligent packaging materials for future use.

## 3. Materials and Methods

### 3.1. Materials, Chemicals and Microbial Strains

Fresh pokeweed (*P. acinosa* Roxb.) berries were harvested from a plantation in Yulin city, Guangxi province (Yulin, China). Fresh shrimps were purchased from a local supermarket (Yangzhou, China). Guar gum (viscosity: ~5500 centipoise), polyvinyl alcohol 1799 (polymerization degree: ~1750), AgNPs (particle size: 60–120 nm, Zeta potential: −25.2 mV, polydispersity index: 0.362), and 2,2-diphenyl-1-picrylhydrazyl (DPPH) were purchased from Macklin Inc. (Shanghai, China). Two bacterial strains including *Listeria monocytogenes* ATCC 19115 and *Salmonella typhimurium* ATCC 14028 were provided by the Food Microbial Lab of Yangzhou University (Yangzhou, China).

### 3.2. Extraction and Characterization of Pokeweed Betacyanins

Betacyanins were isolated from pokeweed berries [24]. Fresh pokeweed berries (220 g) were freeze-dried and ground. The powder (71 g) obtained was extracted overnight in a beaker containing 800 mL of 60% ethanol solution at 4 °C. After filtration, the extract solution was loaded into a chromatograph system (ME99-3, Qingpu Huxi Instrument Inc., Shanghai, China) equipped with a DHL-A pump, DBS-100 fraction collector, HD-3 UV detector and ϕ 1.6 cm × 50 cm column containing AB-8 low polar resin. The chromatograph system was eluted first with distilled water and then with a 60% ethanol solution. The eluates with a red-violet color were collected and vacuum-dried. The dried sample (3.54 g) was stored at −20 °C. The total betacyanin content in the extract powder was 22.67 mg/g powder according to the method of Qin et al. [23]. To test the color-changing capacity of betacyanins, the extract solutions (0.5 mg/mL) with pH adjusted to 3–12 were recorded for their colors and visible spectra at 400–700 nm.

### 3.3. Development of Films

The conventional solvent casting method was used to develop smart packaging films [24]. First, different film-forming solutions with and without AgNPs and PB were prepared. Guar gum/polyvinyl alcohol (GP) solution was obtained by blending 3.4 g guar gum and 1.7 g polyvinyl alcohol in 170 mL hot water (100 °C) for 2 h. The guar gum/polyvinyl alcohol blend solution containing AgNPs (GP-AgNPs) was prepared by adding 0.102 g ultrasonically dispersed AgNPs (2%, *w*/*w*) aqueous suspension into the cooled GP solution. The content of AgNPs (2%, *w*/*w*) was calculated by dividing the whole nanoparticles mass into the total mass of guar gum and polyvinyl alcohol. Guar gum/polyvinyl alcohol blend solutions containing AgNPs and PB (GP-AgNPs-PB) were prepared by adding 0.051, 0.102 and 0.153 g of pokeweed extract powder (1%, 2% and 3%, *w*/*w*) into a cooled GP-AgNPs solution. The content of pokeweed extract powder (1%, 2% and 3%, *w*/*w*) was calculated by dividing the mass of pokeweed extract powder into the total mass of guar gum and polyvinyl alcohol.

All the solutions were added with 1.02 g glycerol (20%, *w*/*w*) as plasticizer and then poured into 24 cm × 24 cm molds. Different films, namely, GP, GP-AgNPs, GP-AgNPs-PB1 (with 1% pokeweed extract powder), GP-AgNPs-PB2 (with 2% pokeweed extract powder) and GP-AgNPs-PB3 (with 3% pokeweed extract powder), were obtained after drying the cast solutions at 20 °C for 72 h. Under this circumstance, the color of the PB was unchanged due to its high stability under neutral conditions. Before the films were characterized, they were equilibrated at 20 °C with 50% relative humidity.

### 3.4. Characterization of Films

#### 3.4.1. Structural Characterization

Film morphology was obtained by observing the cross-section of the film sample with a scanning electron microscope (Gemini 300, Carl Zeiss Inc., Oberkochen, Germany) at 1000× magnification and 2 kV. Intermolecular interactions within the films were determined by recording the infrared spectrum of the film sample with an infrared spectrometer (Varian 670, Varian Inc., Palo Alto, CA, USA) at 400–4000 cm^−1^ with the resolution of 4 cm^−1^. The infrared spectrometer worked in attenuated total reflection mode via 32 scans for each sample.

#### 3.4.2. Color-Related Properties

The film’s initial color was recorded by video microscope (GP-50 HD, Gaopin Instrument Inc., Suzhou, China) and film color values (L*, a*, b* and Δ*E*) were measured by a colorimeter (SR-62, 3NH Technology Inc., Shenzhen, China) [38]. The film’s color-changing capacity was determined under ammonia vapor, which was generated from 13.5 mL of 0.5 mol/L ammonia water [28]. Film color changes under ammonia vapor were recorded by digital camera within 180 min. Film color stability during chilled storage (4 °C, 50% relative humidity) was recorded by digital camera and colorimeter every 3 days over one month.

#### 3.4.3. Barrier Properties

The film light barrier capacity was analyzed by a UV-vis spectrophotometer at 200–800 nm [28]. The film water vapor barrier capacity was analyzed by wrapping the film sample (ϕ 4 cm) around test tube filled with 20 g dried silica gel. The tube was kept at 20 °C in a desiccator (ϕ 30 cm) with 100% relative humidity. Tube weight changes were recorded every day for 5 days [23]. The film’s oxygen gas barrier capacity was analyzed by a gas permeability tester (Basic 201, Labthink Instrument Inc., Jinan, China) at 23 °C with 50% relative humidity [39].

#### 3.4.4. Mechanical Properties

Elongation at break (EAB) and tensile strength (TS) were measured by fixing the film sample (7 cm × 1 cm) on a universal testing machine (STX 200, Yishi Instrument Inc., Xiamen, China) and stretching the film sample at a 1 mm/s speed [39].

#### 3.4.5. Thermal Property

Film thermal degradation behavior was determined by heating the film sample (2.5 mg) from 25 °C to 600 °C on a thermogravimetric analyzer (HTG-1, Henven Instrument Inc., Beijing, China) with 20 mL/min of nitrogen flow as the carrier gas.

#### 3.4.6. Antioxidant Activity

Film antioxidant activity was determined by mixing 1 mL of the film sample solution with 3 mL of 75 μM DPPH methanol solution, keeping the final sample concentration at 1, 2, 3, 4 and 5 mg/mL. The mixture was reacted at 20 °C in a dark environment and measured for its absorbance at 517 nm after 1 h [38].

#### 3.4.7. Antibacterial Activity

The film’s antibacterial activity against *L. monocytogenes* and *S. typhimurium* was determined by the plate colony counting method [40]. In this experiment, nutrient broth agar medium was used to culture the bacteria. Bacterial suspension was first diluted to 10^6^ CFU/mL and then combined with the sterile film sample (2 cm × 1 cm). The mixture was cultured at 37 °C for 24 h. Then, the film antibacterial ratio was determined through plate colony counting, and compared to the control (bacterial suspension without adding film). The antibacterial ratio was calculated by the formula:

(1) $$\mathrm{Antibacterial}\mathrm{ratio}(\%)=\frac{A_{0}-A_{1}}{A_{0}}\times 100$$

where *A*_0_ is the number of clones in control group, and *A*_1_ is the number of clones in the film sample treatment group.

### 3.5. Application of Films

The real-time quality of chill-stored shrimp was indicated by assessing the films via the method of Yao et al. [22]. Fresh shrimp (180 g) was chill-stored in a sterile and transparent box (ϕ 12 cm × 4 cm) with a film sample (2 cm × 1 cm) pasted to the box’s lid. During the chilled storage (4 °C) of shrimp, the real-time quality of the shrimp was tested by measuring its total volatile basic nitrogen (TVB-N) level at 8 h intervals over 48 h. Film color changes were also recorded.

### 3.6. Statistical Analysis

Experimental data were analyzed by ANOVA and Duncan tests. The results are expressed by mean ± standard deviation (SD) and differences were statistically significant at *p* < 0.05.

## 4. Conclusions

Multi-functional food packaging films with good color-changing, barrier, mechanical, antioxidant and antibacterial properties were developed based on a GP matrix, AgNPs and PB. As compared with AgNPs, PB had a more profound impact on reducing the phase-separation of GP and increasing the interactions with the film matrix. The color-changing, barrier, mechanical and antioxidant/antibacterial properties of the GP film were improved by the combined use of AgNPs and PB. The performance of GP-AgNPs-PB films was positively correlated with PB amount, with GP-AgNPs-PB3 showing the best performance. In addition, GP-AgNPs-PB2 and GP-AgNPs-PB3 films effectively indicated shrimp freshness in smart packaging. In the future, GP-AgNPs-PB films could also be used in active packaging to prevent food oxidation and microbial contamination.

## Figures and Tables

**Table 1:** Color parameters of GP, GP-AgNPs and GP-AgNPs-PB films.

Film	L*	a*	b*	Δ*E*
GP	88.86 ± 0.02 ^a^	–0.37 ± 0.11 ^d^	–1.37 ± 0.04 ^a^	4.98 ± 0.73 ^e^
GP-AgNPs	76.27 ± 0.06 ^b^	–0.02 ± 0.03 ^d^	–1.95 ± 0.08 ^a^	13.55 ± 0.06 ^d^
GP-AgNPs-PB1	70.96 ± 0.14 ^c^	11.18 ± 0.14 ^c^	–5.59 ± 0.34 ^b^	23.54 ± 0.06 ^c^
GP-AgNPs-PB2	60.99 ± 0.19 ^d^	24.78 ± 0.25 ^b^	–11.28 ± 0.16 ^c^	40.69 ± 0.12 ^b^
GP-AgNPs-PB3	54.44 ± 0.44 ^e^	34.53 ± 0.39 ^a^	–14.94 ± 0.28 ^d^	52.71 ± 0.15 ^a^

Values are given as mean ± SD (*n* = 3). Different lower case letters in the same column indicate significant difference (*p* < 0.05).

**Table 2:** The TVB-N level of shrimp during chilled storage and the color changes of GP, GP-AgNPs and GP-AgNPs-PB films used to indicate the freshness of shrimp.

Time (h)	TVB-N of Shrimp (mg/100 g)	Color of Films				
		GP	GP-AgNPs	GP-AgNPs-PB1	GP-AgNPs-PB2	GP-AgNPs-PB3
0	5.08 ± 0.21 ^g^	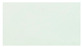	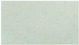	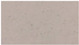	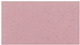	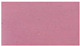
8	9.10 ± 0.28 ^f^	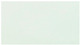	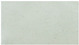	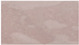	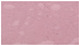	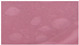
16	12.69 ± 0.31 ^e^	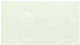	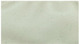	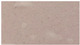	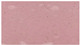	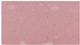
24	16.94 ± 0.15 ^d^	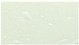	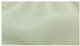	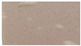	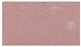	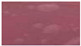
32	23.22 ± 0.11 ^c^	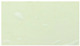	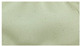	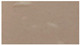	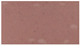	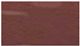
40	32.01 ± 0.23 ^b^	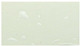	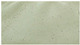	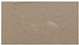	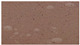	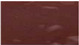
48	46.02 ± 0.16 ^a^	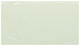	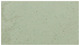	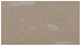	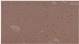	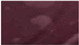

Values are given as mean ± SD (*n* = 3). Different lower case letters in the same column indicate significant difference (*p* < 0.05).

## Data Availability

The data used to support the findings of this study can be made available by the corresponding author upon request.
